# An Opposite-Bending-and-Extension Soft Robotic Manipulator for Delicate Grasping in Shallow Water

**DOI:** 10.3389/frobt.2019.00026

**Published:** 2019-04-24

**Authors:** Zheyuan Gong, Bohan Chen, Jiaqi Liu, Xi Fang, Zemin Liu, Tianmiao Wang, Li Wen

**Affiliations:** ^1^School of Mechanical Engineering and Automation, Beihang University, Beijing, China; ^2^Beijing Advanced Innovation Center for Biomedical Engineering, Beihang University, Beijing, China

**Keywords:** inverse kinematics, soft robotics, underwater robot, soft manipulator, grasping

## Abstract

Collecting seafood animals (such as sea cucumbers, sea echini, scallops, etc.) cultivated in shallow water (water depth: ~30 m) is a profitable and an emerging field that requires robotics for replacing human divers. Soft robotics have several promising features (e.g., safe contact with the objects, lightweight, etc.) for performing such a task. In this paper, we implement a soft manipulator with an opposite-bending-and-extension structure. A simple and rapid inverse kinematics method is proposed to control the spatial location and trajectory of the underwater soft manipulator's end effector. We introduce the actuation hardware of the prototype, and then characterize the trajectory and workspace. We find that the prototype can well track fundamental trajectories such as a line and an arc. Finally, we construct a small underwater robot and demonstrate that the underwater soft manipulator successfully collects multiple irregular shaped seafood animals of different sizes and stiffness at the bottom of the natural oceanic environment (water depth: ~10 m).

## Introduction

**Figure 1:**
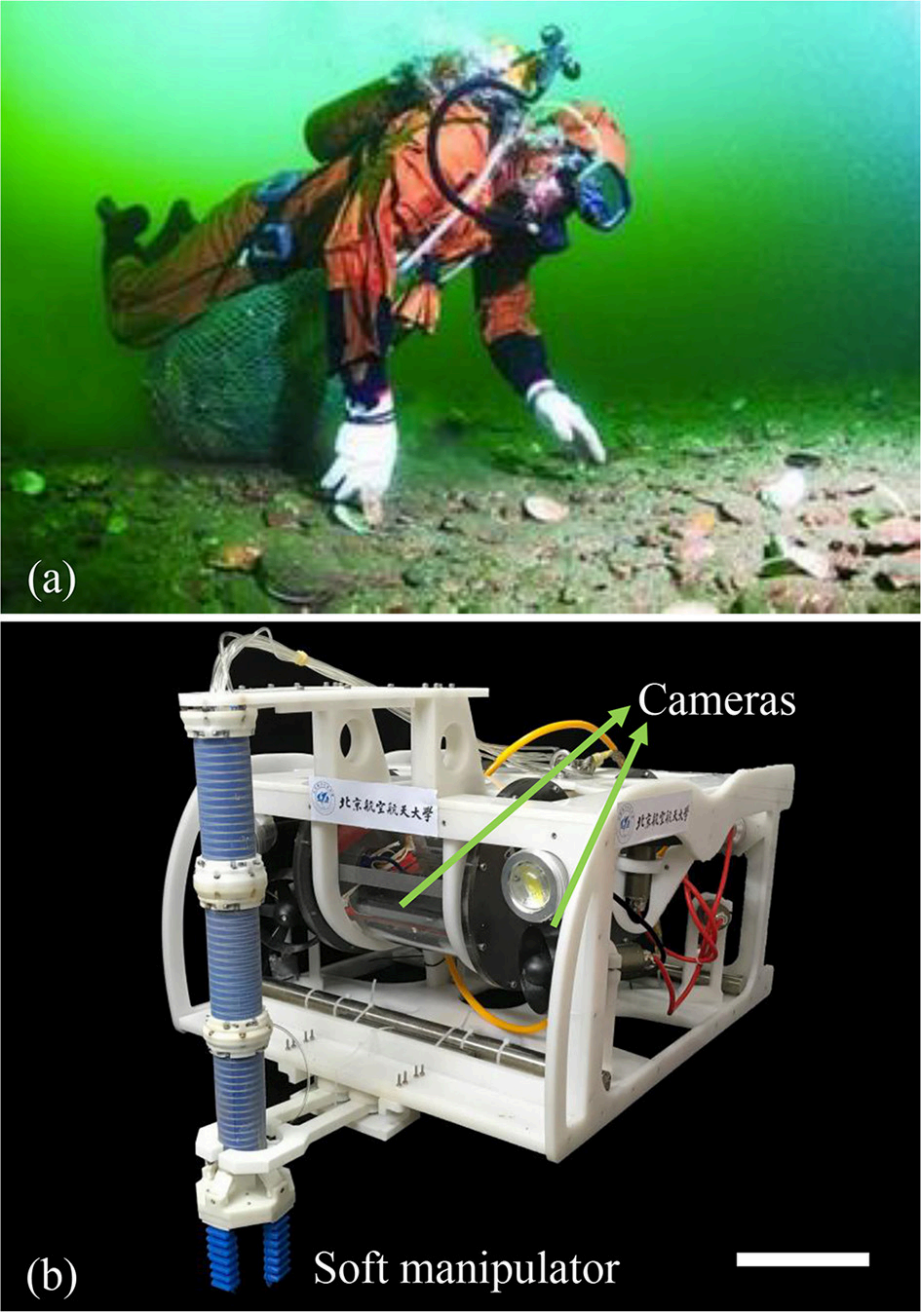
**(a)** Seafood collection by a human diver. **(b)** The snapshot of the underwater robot with a soft manipulator for grasping fragile sea animals. Multiple cameras are applied to provide underwater vision. The length of scale bar is 100 mm.

Collecting seafood animals cultivated in the shallow water is a promising industry, which requires growing autonomic and robotic technologies. Traditionally, human divers are assigned to manually collect the seafood animals such as sea cucumbers, sea echini, scallops, etc. (Figure 1a). However, long-time working under the water depth of 10–30 m would cause the divers suffering from severe occupational disease including rheumatism, gout, osteonecrosis, etc. Collecting seafood animals in the harsh, shallow water environment requires small underwater robot and flexible manipulation system. Previously, the rigid robotic arms used for underwater manipulation have several challenging issues such as delicate grasping fragile and squishy seafood animals. Meanwhile, the traditional rigid hydraulic arms usually have large mass. The huge inertia caused by the rigid arm during locomotion would induce significant vibration for the small underwater vehicle (Fernandez et al., 2013).

Soft robots provide an alternative way to collect these fragile sea animals, due to the properties of compliance and safe interaction. Recently, increasing studies on soft robotics have focused on the underwater applications. For example, robotic octopus arms achieved underwater locomotion (Calisti et al., 2011; Cianchetti et al., 2015); soft gripper has been used for biological sampling the coral reefs (Galloway et al., 2016); the origami gripper was applied to collecting delicate midwater organisms (Teoh et al., 2018); the jamming gripping was exploited in handling in deep sea (Licht et al., 2017); a soft glove was integrated to tele-operated control the soft wrist modules for biological underwater grasping (Kurumaya et al., 2018; Phillips et al., 2018).

Previously, the piecewise constant curvature (PCC) model has been developed (Webster Iii and Jones, 2010) and is used for modeling the flexible continuum robots (Webster et al., 2007) and soft elastomeric arms (Gong et al., 2017, 2018a,b), etc. The inverse kinematics modeling is another challenge issue. To address this challenge, previous studies have regarded the continuum joint as 3UPS-1PU-extensible structure for simplification and further developed the DH method for inverse kinematic modeling of flexible manipulator (Lakhal et al., 2014); Jacobian iteration was applied to determine the inverse kinematics for the underwater soft manipulator in the two-dimensional space (Marchese and Rus, 2016); machine learning algorithms were proposed to train a single soft actuator (Giorelli et al., 2015; Lee et al., 2017) and a two-dimensional soft manipulator (Jiang et al., 2017). Natural-CCD algorithm was proposed to generate simple, precise, and computationally efficient inverse kinematics (Martín et al., 2018). However, previous studies have not yet experimentally explored the spatial manipulation with inverse kinematics, particularly for the collecting tasks in natural underwater environment.

To complement a controllable underwater soft robotic manipulator for seafood grasping in shallow water, in this paper, we propose a novel inverse kinematic method. Based on an opposite-bending-and-extension structure of the robotic arm, our method enables point-point movements in three-dimensional space and trajectory planning. We mount the underwater soft manipulator on a small underwater vehicle and then demonstrate underwater picking and placing seafood animals. Our current study shows wide-open applications of soft robotic manipulator in the shallow water undersea environment.

## Materials and Methods

### System Overview

Soft robots have intrinsic compliance, which have significant advantages for grasping these seafood animals (for instance, sea cucumber has a modules of ~10^6^Pa). In order to implement the underwater grasping in shallow water, we construct a small underwater robot with a soft manipulator (modules around ~10^5^Pa), as shown in Figure 1b. The underwater soft manipulator can achieve 3-DOF movement and grasping. A 4-DOF underwater remotely operated vehicle (ROV) is integrated with two cameras, one of which is for grasping from near top view, while another is for guiding movement from large side view. Through live cameras, both the underwater soft manipulator and ROV are remotely controlled by the human operator on a boat. The underwater soft manipulator is 360 mm in length (300 mm for only the soft arm) and 34 mm in diameter, with a total mass of 322 g. The robot measures 600 mm long, 500 mm width, and 300 mm tall, with a weight of 10 kg, and operated depth of 50 m.

### The Underwater Soft Manipulator

**Figure 2:**
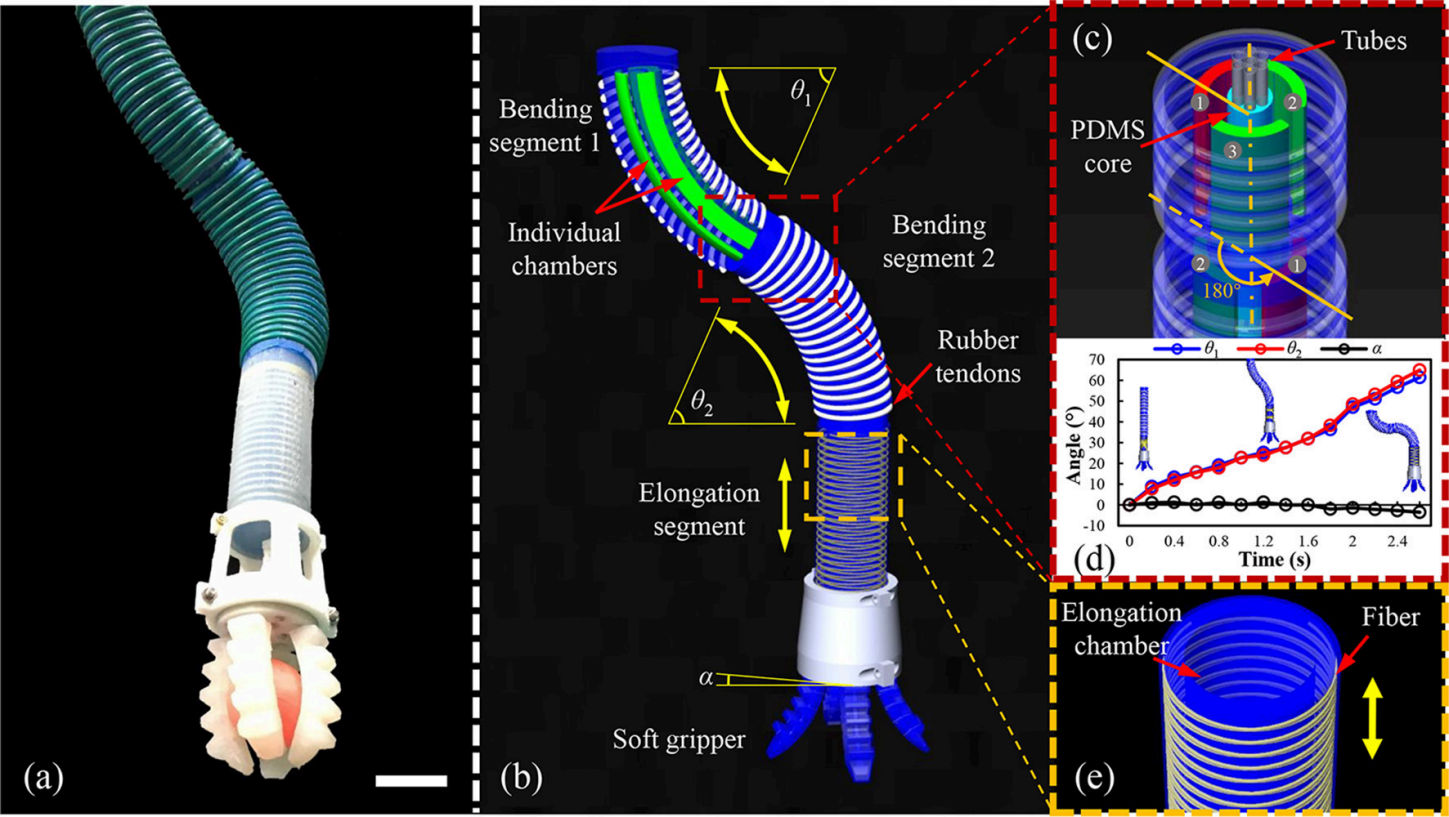
The design and principle mechanics of the underwater soft manipulator. **(a)** An overall side image of the underwater soft manipulator (scale bar 50 mm). **(b)** The underwater soft manipulator is applied modularized design that consisted of two bending segments, an elongating segment, and a soft gripper. θ_1_ and θ_2_ represent the bending angles of the two bending segments, and α represents the horizontal angle of the manipulator tip. The manipulator is actuated with an opposing curvature where θ_1_ = θ_2_ and α = 0. **(c)** The two bending segments had a joining angle of 180°. **(d)** θ_1_, θ_2_, and α are verified in one actuation with opposing curvature. The two bending angles (θ_1_, θ_2_) are almost equal, and the horizontal angle (α) is zero at each moment. **(e)** The fiber-reinforced elongating segment. The yellow arrow indicates the direction of elongation.

We design and fabricate an entirely soft, underwater manipulator with soft gripper as the end effector (Figure 2a) (Martinez et al., 2013; Polygerinos et al., 2015; Hao et al., 2018). The underwater soft manipulator consists four sections: two bending segments, one elongation segment, and one soft gripper (Figure 2b). This bending segments and elongation segment are designed to have a circle shaped cross-section to decrease the hydrodynamic resistance in the water flow (Gong et al., 2018b). Each bending segment has three individual chambers. Meanwhile, it is covered with rubber tendons to reduce radial ballooning of the chambers when pressurizing (Figure 2c). We apply the fiber-reinforced actuator on the elongation segments to provide extension in the vertical direction while grasping underwater (Figure 2e).

Note that we actuate the underwater soft manipulator in a special manner to simplify the kinematic modeling (will introduce later): the two bending segments are actuated with the same bending curvature but opposite bending direction. We regard that the kinematics is established on this opposite-bending-extension actuation condition. We integrate two bending segments at an included angle of 180° (Figure 2c). When we actuate the opposite chambers of the two bending segments, the underwater soft manipulator always perform the opposite-bending-extension condition even in the spatial space. Besides, the air pressures in the opposing chambers have linear relationship, which means only one pressure is required for the kinematics model. Under this linear relationship, the curve angles of two bending segments (θ_1_, θ_2_) are almost the same and the intersection angle at the horizontal level of the underwater soft manipulator tip (α) is zero, which is the soft gripper is always facing vertically down to the ground. Figure 2d shows θ_1_, θ_2_, and α in one trial when actuating the underwater soft manipulator. We find that the values of θ_1_, θ_2_, and α confirm the design to realize opposing curvature.

### Kinematics Modeling

**Figure 3:**
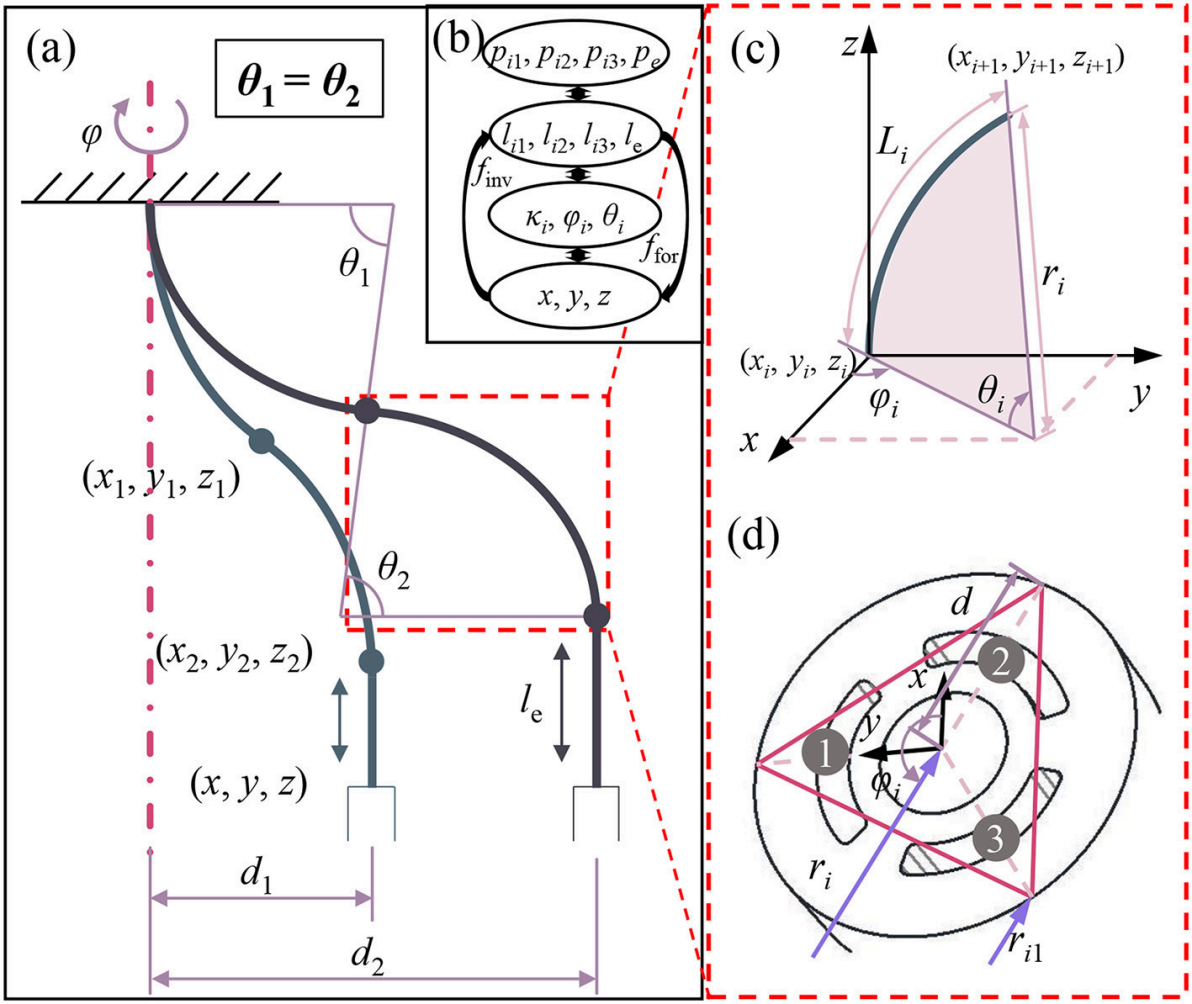
Kinematics of the underwater soft manipulator. **(a)** The two bending segments of the underwater soft manipulator are always actuated with opposing curvatures. Thus, the bending angles are always equal (θ_1_ = θ_2_). **(b)** The kinematic transformation map. We focused on inverse kinematics (*f*_inv_) from the position parameters (*x*_*i*_*, y*_*i*_*, z*_*i*_) to chamber lengths (*l*_*i*__1_*, l*_*i*__2_*, l*_*i*__3_*, l*_*e*_) via arc parameters (κ_*i*_, φ_*i*_, θ_*i*_). **(c)** Geometric schematic in a bending segment, where φ_*i*_ represents the rotation angle around the *z*-axis; θ_*i*_ represents the bending angle around the *y*-axis; *r*_*i*_ represents the radii of the bending curve; and *L*_*i*_ represents the centerline length of the bending segment. **(d)** Geometric schematic of the cross-section, where *h* represents the distance between the arm surface and the center of the cross-section.

Figure 3a demonstrates the kinematics of the underwater soft manipulator. With opposite-bending-extension condition, the two bending segments shared the degrees of freedom (DOF) only have 2 DOF (x-y plane), while one bending segment has the DOF of the rotation φ and bending θ_1_ (or θ_2_). Due to the elongation segment (z-axis), we can achieve three DOF movements and grasping.

Opposing curvature patterns offers plenty of advantages on kinematic modeling. First, the attitudes of two bending segments {κ_*i*_, φ_*i*_, θ_*i*_} (κ_*i*_ refers to the bending curvature of the *i*th segment) have very straightforward relationships (Equations 4–6). And we only need to calculate the attitudes of one segments. Second, it reduces the number of inputs (seven independent chambers totally to four independent chambers). Thus, it reduces half of the computation contributed to the fast solution of kinematics.

The modeling procedure can be realized in two parts (Figure 3b): (1) the transformation between the coordinates of the end effector {*x, y, z*} and chambers length {*l*_*i*1_*, l*_*i*2_*, l*_*i*3_*, l*_*e*_} (*l*_*ij*_, the indexes *i* and *j* mean segment *i* chamber *j*; *l*_*e*_, the index *e* means the length of elongation segment; the same as below). The essential point of this part is how we can get an inverse solution from {*x, y, z*} (three parameters) to {*l*_*i*1_*, l*_*i*2_*, l*_*i*3_*, l*_*e*_} (four parameters) without other inputs. (2) The transformation between chambers length {*l*_*i*1_*, l*_*i*2_*, l*_*i*3_*, l*_*e*_} and the pressure {*p*_*i*__1_, *p*_*i*__2_, *p*_*i*__3_, *p*_*e*_}, the directly actuation parameter. By reason of the nonlinear response of soft material and complexity of structures, it is complicated to figure out (2) in a theoretical way, so we finish this work via experiments (**Figure 5**), and we fit formulas for the model-based control recording these results. In order to simplify the kinematics model, we make the following assumptions:

- The bending section have the constant curvature rate, and the elongation section is straight. The curves are tangent at the intersection points.
- The chambers in the same segment are parallel, and the cross sections are equal in the same section.

#### Forward Kinematics

Previous studies have already shown how to solve the forward transformation questions (Webster Iii and Jones, 2010; Gong et al., 2017). Combining these methods with the structures and sizes of our soft arm (shown in Figure 3c), we can obtain coordinates of the segments tip {*x, y, z*} from the length of the chamber {*l*_*i*1_*, l*_*i*2_*, l*_*i*3_*, l*_*e*_} with the help of attitudes {κ_*i*_, φ_*i*_, θ_*i*_}.

(1) $$\begin{array}{lll} \kappa_{1} & = & \frac{1}{r_{1}}=\frac{2\sqrt{l_{11}^{2}+l_{12}^{2}+l_{13}^{2}-l_{11}l_{12}-l_{11}l_{13}-l_{12}l_{13}}}{\left(l_{11}+l_{12}+l_{13}\right)d} \end{array}$$

(2) $$\begin{array}{lll} \varphi_{1} & = & \tan^{-1}\left(\frac{l_{12}+l_{13}-2l_{11}}{\sqrt{3}\left(l_{12}-l_{13}\right)}\right) \end{array}$$

(3) $$\begin{array}{lll} \theta_{1} & = & \frac{2\sqrt{l_{11}^{2}+l_{12}^{2}+l_{13}^{2}-l_{11}l_{12}-l_{11}l_{13}-l_{12}l_{13}}}{3d} \end{array}$$

In equations (1–3), *d* represents the radius of soft arm cross-section, and *r*_1_ is the radius of the bending curve. Particularly, we use the surface length to represent the chamber length mainly considering it is more accessible for measurement. After we got the attitudes parameters from the bending segment 1, we can get attitudes of the bending segment 2:

(4) $$\begin{array}{lll} \kappa_{2} & = & \kappa_{1} \end{array}$$

(5) $$\begin{array}{lll} \varphi_{2} & = & \varphi_{1}+\pi \end{array}$$

(6) $$\begin{array}{lll} \theta_{2} & = & \theta_{1}\; \end{array}$$

Furthermore, we can also get the coordinate of soft arm tip {*x, y, z*} from the attitudes {κ_*i*_, φ_*i*_, θ_*i*_} we got previously. Mathematically, we consider the underwater soft manipulator simply consisted of constant curvature curves (bending segments) and lines (elongation segments) based on the assumptions. The coordinate transformation in both curves and lines can be described by homogeneous matrixes shown in equation (7), where *R* is the rotation matrix, and *p* is the translation vector.

(7) $$\begin{array}{l} \text{T}={\left[\begin{array}{cc} R & p\\ 0 & 1 \end{array}\right]}_{4\times 4} \end{array}$$

Figure 3c shows the modeling of a single segment. We define orientation angle φ_*i*_ represents the rotation angle around the *z*-axis, curvature angle θ_*i*_ represents the bending angle around the *y*-axis, where *i* indicates the *i*th segment. In the bending segments, we consider the bending procedure as: first the soft arm rotates around *y*-axis with angle θ_*i*_; second, the soft arm rotates around *z*-axis with angle φ_*i*_. Moreover, we need to post-multiply the homogeneous matrix with the rotation matrix *R*(-φ_*i*_) and zero translation. The transformation matrix for the bending segment is demonstrated in equation (8) In elongation segments, we only need to consider the translation on *z*-axis with a length of *l*_*e*_ (Equation 9).

(8) $${\;}_{i-1}^{i}T=\left[\begin{array}{cc} R_{z}\left(\varphi_{i}\right) & 0\\ 0 & 1 \end{array}\right]\;\cdot \;\left[\begin{array}{cc} R_{y}\left(\theta_{i}\right) & 0\\ 0 & 1 \end{array}\right]\;\cdot \;\left[\begin{array}{cc} R_{z}\left(-\varphi_{i}\right) & 0\\ 0 & 1 \end{array}\right]=\left[\begin{array}{cccc} cos^{2}\varphi_{i}\cos\theta_{i}+sin^{2}\varphi_{i} & \cos\varphi_{i}\sin\varphi_{i}\left(\cos\theta_{i}-1\right) & \cos\varphi_{i}\sin\theta_{i} & r\cos\varphi_{i}\left(1-\cos\theta_{i}\right)\\ \cos\varphi_{i}\sin\varphi_{i}\left(\cos\theta_{i}-1\right) & sin^{2}\varphi_{i}\cos\theta_{i}+cos^{2}\varphi_{i} & \sin\varphi_{i}\sin\theta_{i} & r\sin\varphi_{i}\left(1-\cos\theta_{i}\right)\\ -\cos\varphi_{i}\sin\theta_{i} & -\sin\varphi_{i}\sin\theta_{i} & \cos\theta_{i} & r\sin\theta_{i}\\ 0 & 0 & 0 & 1 \end{array}\right]$$

(9) $${\;}_{2}^{3}T=\left[\begin{array}{cccc} 1 & 0 & 0 & 0\\ 0 & 1 & 0 & 0\\ 0 & 0 & 1 & l_{e}\\ 0 & 0 & 0 & 1 \end{array}\right]$$

Thus, we can get the forward transformation of the whole soft manipulator (Equation 10).

(10) $${\;}_{0}^{3}T{=}_{0}^{1}T\;\cdot {\;}_{1}^{2}T\;\cdot {\;}_{2}^{3}T$$

#### Inverse Kinematics

With this inverse kinematics method, we can realize the coordinate based control and point to point movement of the underwater soft manipulator. That is the foundation of the picking and placing tasks, as well as the trajectory planning. Further, the quick solution of inverse kinematics also helps to improve the real-time control ability of soft manipulator. However, the inverse kinematics of soft robots (even continuum robots) is always a challenging problem (Webster Iii and Jones, 2010). The large group's nonlinear equations in the transformation matrix cause the huge complexity to the inverse solution.

We propose a rapid inverse solution on soft manipulators with the specific opposite-bending-extension condition. As we discuss above that the underwater soft manipulator has three DOF in coordinate space {*x, y, z*}. However, the underwater soft manipulator has four independent chambers {*l*_*i*1_*, l*_*i*2_*, l*_*i*3_*, l*_*e*_}. In order to get the chambers length {*l*_*i*1_*, l*_*i*2_*, l*_*i*3_*, l*_*e*_} (four outputs) from the coordinates {*x, y, z*} (three inputs), we propose a constraint condition: at most two chambers in a bending segment are actuated at the same time, so that at least one chamber in one bending segment is in initial length. Thus, the point of this method is to figure out which chamber is in initial length.

We also resolve the transformation from {*x, y, z*} to {*l*_*i*1_*, l*_*i*2_*, l*_*i*3_*, l*_*e*_} with the attitudes {κ_*i*_, φ_*i*_, θ_*i*_}. First, we obtain the rotation angle φ_1_ from the given inputs {*x, y, z*}.

(11) $$\begin{array}{l} \varphi_{1}=-\tan^{-1}\left(\frac{y}{x}\right) \end{array}$$

Then we evaluate φ_1_ to figure out which two chambers need to be actuated. According to the geometry relationship in Figure 3d, we can give an equation where we represent the initial length with the attitudes parameters {κ_*i*_, φ_*i*_, θ_*i*_}. The initial length of chambers can be pre-measured by camera calibration. Here, on the relationship κ_*i*_ = *r*_*i*_^−1^, we also regarded *r*_*i*_ as attitudes parameter κ_*i*_.

(12) $$\begin{array}{l} {\;}_{0}^{3}T{=}_{0}^{1}T\;\cdot {\;}_{1}^{2}T\;\cdot {\;}_{2}^{3}T\\ \{\begin{array}{l} l_{i1\text{init}}=\theta_{1}\;\cdot \;\left(r_{1}-d\sin\varphi_{1}\right),when\frac{\pi }{6}\leq \varphi_{1}<\frac{5\pi }{6}\\ l_{i2\text{init}}=\theta_{1}\cdot \left[r_{1}+d\cos\left(\varphi_{1}-\frac{\pi }{6}\right)\right],when\frac{5\pi }{6}\leq \varphi_{1}<\frac{3\pi }{2}\\ l_{i3\text{init}}=\theta_{1}\cdot \left[r_{1}-d\cos\left(\varphi_{1}+\frac{\pi }{6}\right)\right],when\frac{3\pi }{2}\leq \varphi_{1}<2\pi \;or0\leq \varphi_{1}<\frac{\pi }{6} \end{array} \end{array}$$

Considering the geometry relationship shown in Figure 3D, we derive another equation from the given coordinate:

(13) $$\begin{array}{l} \frac{x}{2}=r_{1}\cdot \cos\varphi_{1}\cdot \left(1-\cos\theta_{1}\right) \end{array}$$

In equations (12) and (13), we can found that only *r*_1_ and θ_1_ are the unknown quantities. Combining the two equations, we can solve the rest attitudes parameters. Then, we easily obtained the length of all chambers {*l*_*i*1_*, l*_*i*2_*, l*_*i*3_*, l*_*e*_}.

(14) $$\{\begin{array}{l} l_{i1}=\theta_{i}\left(r_{i}-d\sin\varphi_{i}\right)\\ l_{i2}=\theta_{i}\left[r_{i}+d\cos\left(\varphi_{i}-\frac{\pi }{6}\right)\right]\\ l_{i2}=\theta_{i}\left[r_{i}-d\cos\left(\varphi_{i}+\frac{\pi }{6}\right)\right]\\ l_{e}=-2r_{1}\sin\theta_{1}-z \end{array}$$

**Figure 4:**
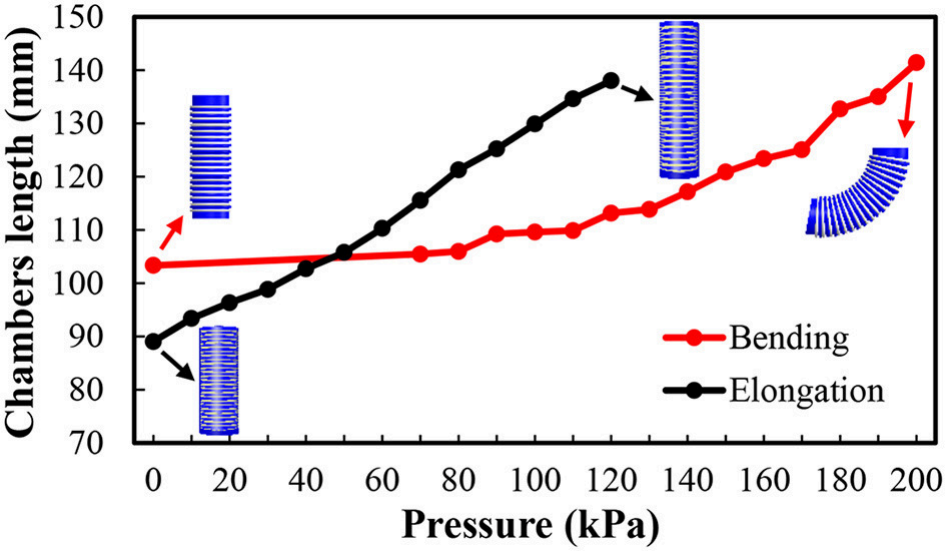
The multi-channel pneumatic actuation system for the underwater soft manipulator. The system contains a microcontroller, power source, DA converter, ten proportional valves, and ten air pressure sensors.

According to above equations, we obtain specific inverse transformation from {*x, y, z*} to {*l*_*i*1_*, l*_*i*2_*, l*_*i*3_*, l*_*e*_}. With the help of the pressure – length calibration (Figure 4), we can further transfer from {*l*_*i*1_*, l*_*i*2_*, l*_*i*3_*, l*_*e*_} to the actuating pressure {*p*_*i*__1_, *p*_*i*__2_, *p*_*i*__3_, *p*_*e*_} for our model-based pneumatic control.

### Actuation and Control

**Figure 5:**
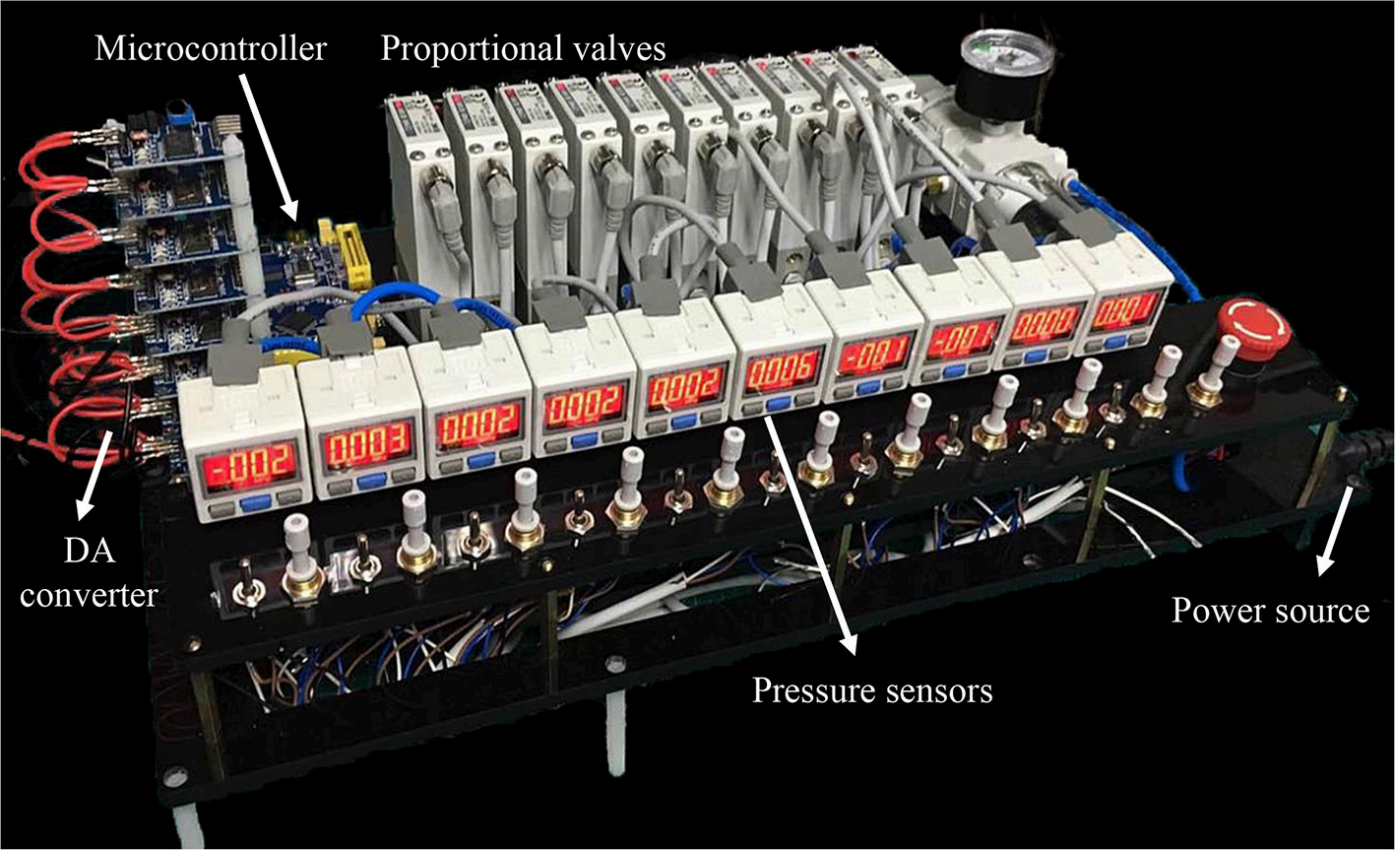
Chamber lengths of the bending segments (red) and elongating segment (black) as a function of the actuation pressure.

The current soft manipulator is actuated by pneumatic pressure. We implement a multi-channel pneumatic driving system, shown in Figure 5. The system has ten pneumatic channels while each channel can generate pressure independently with maximum of 500 kPa to actuate the chambers in the underwater soft manipulator. The system contains a microcontroller (STM32F103, *STMicroelectronics*, Italy and France), DA convertors (PCF8591, *NXP*, Netherland), proportional valves (ITV0030-2BL, *SMC*, Japan), pressure sensors (ISE30A, *SMC*, Japan), an air compressor, and related software. We apply PID method in the closed-loop control of pressure, which is continuously adjusted according to the data from pressure sensors.

To control the underwater soft manipulator, we program the inverse-kinematics-model-based control algorithm in MATLAB. Calculating pressures from the reference coordinates, the software can conduct the underwater soft manipulator to pick and place object at specific positions. The calculated pressures can be sent to actuation system via RS232 communication protocol. By dividing the trajectory path into small segments (0.5 mm) and inserting desired points, we can control the underwater soft manipulator to follow a specific trajectory. In the field application, we also balance the pressures in chambers of the underwater soft manipulator according to the water depth. The balance transformation is shown in the equation (15), where *p*_d_ is the pressure applied, *p*_0_ is the pressure calculated, ρ_environment_ is the underwater environment density (1025 kg/m^3^ is considered as the sea water density), *h*_d_ is the depth where the robot works. It should be noted that the underwater soft manipulator is mainly designed for grasping fragile sea animals, which most of them are suspending in the water and have no load on the underwater soft manipulator. Currently, we have not considered the influence of the gravity and loads on control of the underwater soft manipulator.

(15) $$\begin{array}{l} p_{d}=p_{0}+\rho_{environment}gh_{d} \end{array}$$

### Laboratory Experiments Setup for Characterizing the Underwater Soft Manipulator

In order to evaluate the capability of the kinematic model, we perform experiments on the model based location error and trajectory planning. We apply a stereo cameras system to capture the motions and trajectories. The underwater soft manipulator is mounted in water and actuated by the multi-channel pneumatic system. The stereo cameras is carefully calibrated, and the error is less than 0.5 mm. Moreover, we rebuild the motions and got the coordinates of marker points from the images of different views. We perform the location error in different directions (φ_i_) with the distance (*d*) ranging from 0mm to 100 mm, 10 mm of step length. We also perform the trajectory planning ability with paths of line and circle. Then, we run the workspace simulation in MATLAB.

## Results

### Kinematic Model Validation, Trajectory Planning, and Workspace Simulation

**Figure 6:**
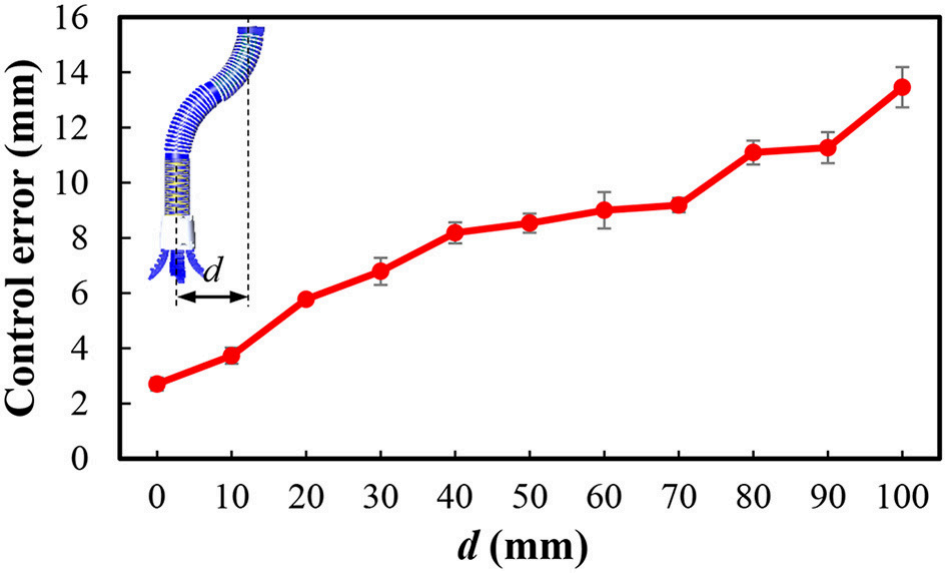
Control location error as a function of operating radius *d* (0 to 100 mm).

**Figure 7:**
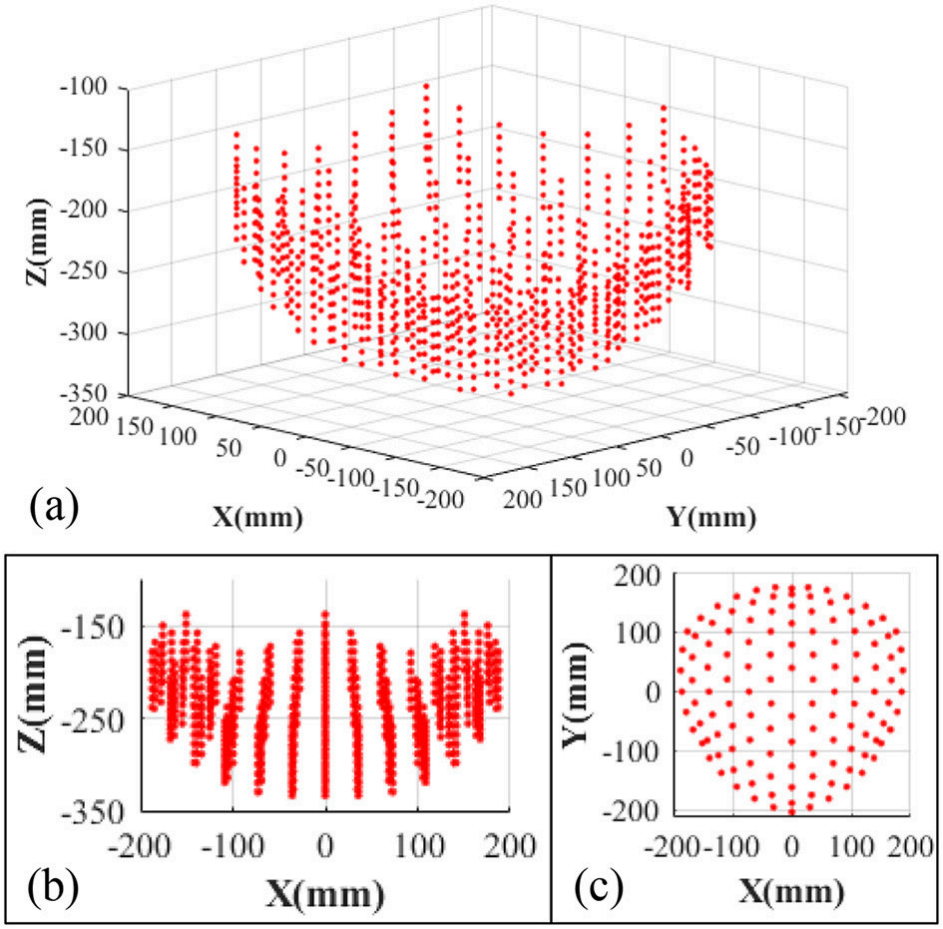
**(a)** The simulation of operating workspace of the underwater soft manipulator. **(b)** The front view of the workspace. **(c)** The top view of the workspace.

The underwater soft manipulator is actuated to move different distances (*d*), and the average control errors (between the experiments and simulations) of both the distances and rotational angles of the manipulator's base (φ_i_) are demonstrated in Figure 6. We found that the errors are within the range of 2.7~13.4 mm when the distances changing from 0mm to 100 mm. This error range is tolerant to the soft gripper while grasping [the tolerant deviation of gripper and objects that led to successful grasp (Hao et al., 2018)]. According to the kinematic model, simulation on the workspace of the soft arm is illustrated as Figure 7. The results show that the underwater soft manipulator collected a plate-shaped workspace with a size of approximately 400 mm in diameter and 100 mm height.

**Figure 8:**
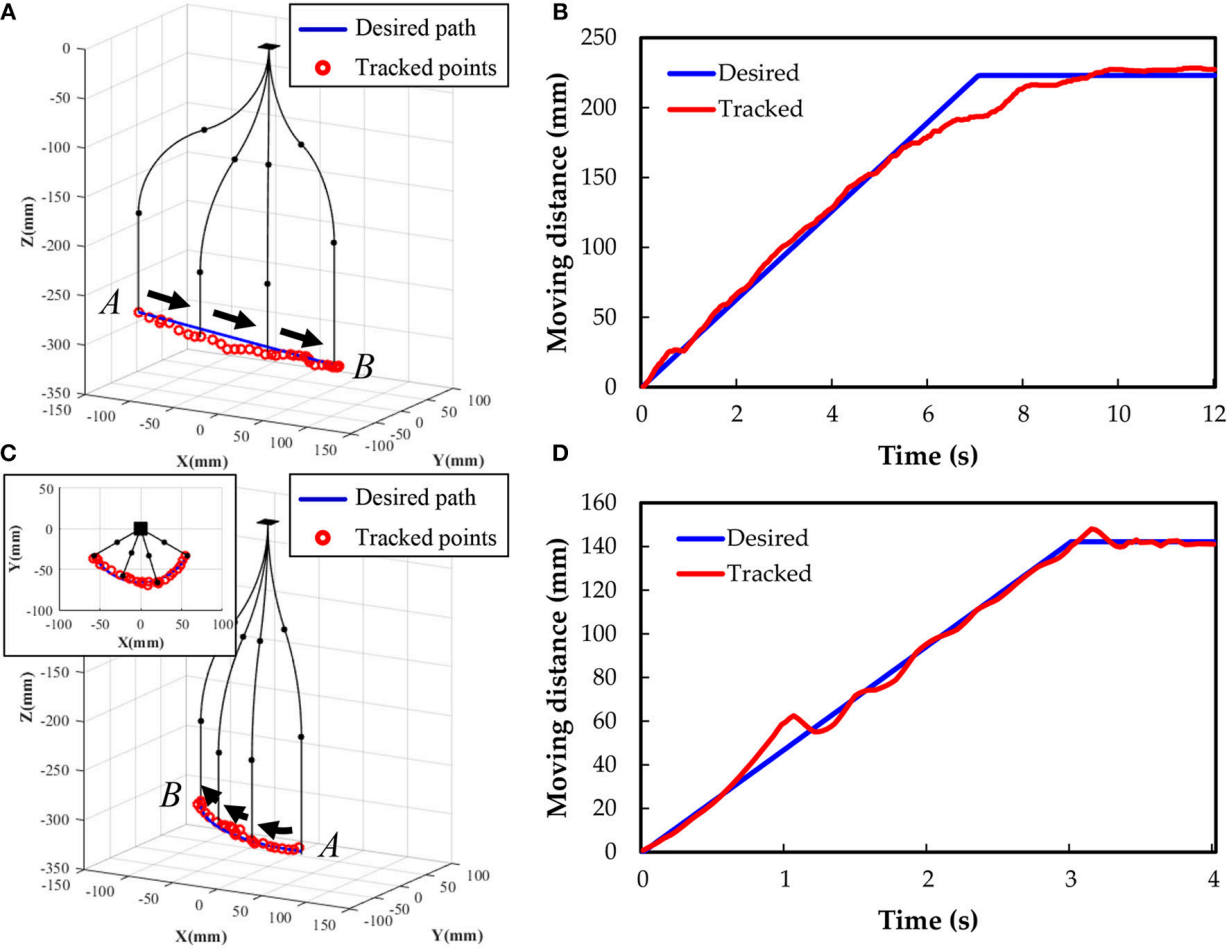
Controlled soft manipulator following trajectories of a line **(A)** and an arc **(C)**. Distance response with time while tracking **(B)** the line trajectory and **(D)** the arc trajectory.

Furthermore, we demonstrate the trajectory planning ability of the underwater soft manipulator with paths of line shaped and arc shaped trajectories (Figure 8). While performing the line trajectory (Figure 8A), the underwater soft manipulator is actuated from the point A (−110, −64, −270) (unit: mm) to the point B (110, 64, −295) at a programmed speed of 32 mm/s. The red circles are tracked points from the experiments; the blue lines are the computer-programmed path. The black lines represent the underwater soft manipulator, and the black dots on the black lines represent the intersections of different segments. The results show that experiment trajectory has a small error from the desired path in 3D space. The tracked points match the programmed path well and the error is less than 6.6 mm (Figure 8B). In the arc shaped trajectory, the underwater soft manipulator is actuated from the point A (−55, −35, −285) to the point B (55, 35, −320) with a rotation angle of 120°, radius of 65 mm and programmed speed of 45 mm/s, as shown in Figure 8C. We observe a vibration when suddenly changed moving direction of the underwater soft manipulator (Figure 8D). Lines and arcs are the fundamental shapes of trajectory; therefore, we hope more complex trajectory tracking can be achieved in the future based on the current work.

### Field Test of Underwater Grasping

**Figure 9:**
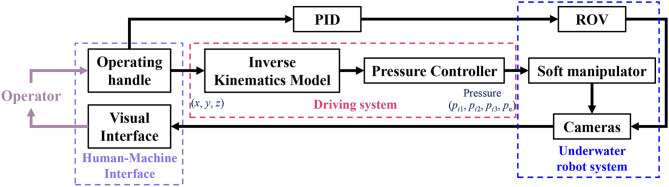
System architecture of the underwater grasping robot. The system contains human-machine Interface, driving system and the underwater robot system. The underwater robot is operated to approach the target seafood animals by the human operator. Then the underwater soft manipulator is controlled pick and place the sea animals via inverse kinematics model. The whole process is monitored by cameras which facilitates the remote control of human operator.

To examine the capabilities of the underwater soft manipulator, we construct an underwater robot with the underwater soft manipulator (Figure 1b), and perform the underwater grasping of fragile marine seafood animals (e.g., sea cucumbers, echini, etc.) in the natural undersea environment. A 4-DOF underwater vehicle is integrated with two cameras, which provide images from near top view for the grasping and large side view for the movement guiding. The movements of the underwater vehicle is under PID control that enable stable swimming and hovering. The underwater robot is powered from a ship floating above the grasping area. Both the underwater soft manipulator and underwater vehicle are under remote control via the real-time underwater cameras (transmitting images via cables). Figure 9 shows the system architecture applied for the undersea grasping, which is realized in three main steps: (1) The underwater robot is operated to approach the targets area and performs hovering and searching the seafood animal targets. (2) The underwater robot sinks to the bottom of the ocean. Then the underwater soft manipulator is controlled via inverse kinematics model to approach the undersea animals with the soft gripper open. (3) The underwater soft manipulator picks the target and places it into the collecting basket. While working underwater, the environment pressure is variable in different operating depth. Thus, the actuating pressures in chambers of the underwater soft manipulator are balanced according to the depth change (see Equation 15).

**Figure 10:**
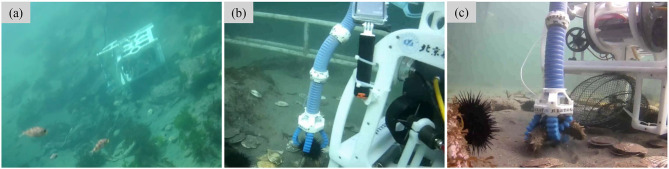
Undersea grasping with the underwater soft manipulator mounted on a small underwater robot. **(a)** The underwater grasping is demonstrated in the natural undersea environment at 10 m depth. **(b,c)** Grasping undersea animals (echini and sea cucumbers) with soft manipulator.

Figure 10 shows the field grasping in the natural undersea environment (Figure 10a), where the depth is 10 m and the speed of current in the ocean bottom is about 2 m/s. The seabed is covered by sand and stones, and the animals spread around and even partially embedded in sand and rocks. Finally, we successfully grasp echini, sea cucumbers, and shells at the depth 10 m undersea within 20 min (Figures 10b,c).

## Conclusion and Discussion

In this paper, we construct a soft manipulator and ROV system for seafood grasping in shallow water. The underwater soft manipulator is designed and actuated as opposite-bending-extension condition and can achieve 3-DOF movements in space. It is controllable with a simple but rapid inverse kinematics. The results show that the error is less than 13.4 mm, and we achieve the trajectory planning by tracing the paths of a line and an arc. The experimental tracking points fit the desired position well in both positions and timescale. Finally, we perform the field test—we tested the manipulation capacity of soft arm in the natural undersea environment. The soft arm manipulator successfully grasp sea animals at a sea depth of 10 m via an underwater robot. Hopefully, this robot can be used for dexterous grasping in shallow water environment (0–50 m), and can replace the human divers for safely harvesting the seafood efficiently, without any damage.

We propose a simple and universal inverse solution for the underwater soft manipulators whose structure and actuation are followed the opposite-bending-extension condition. Distinct from the previous D-H method (Lakhal et al., 2014), machine learning model (Giorelli et al., 2015; Lee et al., 2017), and Jacobian iteration (Marchese and Rus, 2016), this inverse kinematics can be applied for the whole manipulator and does not require heavy computational resources, which enables real-time control in application. This method has also been tested and validated at the natural oceanic environment. The results have proved that reducing the DOFs of the soft manipulator is a possible approach to solve the inverse kinematics problem. The underwater soft manipulator with inverse kinematics can operate in the natural unstructured undersea environment without precise kinematic and force sensory feedback as the rigid manipulators do. Furthermore, the rigid robotic arms and grippers for the underwater manipulations have a huge mass and inertia which impacts their maneuverability. In contrast, soft robots have advantages of compliance and lightweight and may play an important role in underwater manipulation. Compared with the rigid hydraulic manipulators, our soft manipulator has exceptional features of lightweight and low inertia. The underwater soft manipulator has a mass of 0.322 kg (almost zero mass in water), while with a length of 360 mm. The current prototype is significantly lighter than the traditional rigid hydraulic manipulators that commonly has a mass of tens of kilograms, e.g., a hydraulic manipulator with a length of 499 mm has a total mass of 17.2 kg (Fernandez et al., 2013). Thus, locomotion of the underwater soft manipulator has negligible inertial effect for the underwater vehicle than the traditional rigid underwater manipulator.

Previous studies have shown the promising features of soft robots for the deep sea application (Calisti et al., 2011; Cianchetti et al., 2015; Galloway et al., 2016; Licht et al., 2017; Kurumaya et al., 2018; Phillips et al., 2018; Teoh et al., 2018). In this paper, we demonstrate a soft manipulator system with dexterous motions, which aims for the shallow water seafood animal grasping (sea cucumbers, echini, etc.). In the 10 m depth natural, undersea environment, our soft manipulator showed controllable motions under the inverse kinematic model. It can be remotely controlled to pick and place at the specific location coordinated with the underwater cameras, and we achieve more than 80% of succession rate of grasping multiple irregular shaped objects of different sizes and stiffness. Our results show that the underwater soft manipulator has inherent advantages of compliance and is promising for the future underwater manipulation. In addition, the multi-channel pneumatic actuation system and pressure balancing method (equation 15) plays significant roles in the real-world underwater grasping. Thanks to the pressure balancing method, the pressure differential inside and outside of the chambers can be maintained as constant. As a result, the underwater soft manipulator is able to achieve almost identically motions in different operating depth and collect seafood animals in the natural unstructured environment.

In this study, the inverse kinematics method reduce the DOFs to only three. Taking into account the control of the spatial angles of the manipulator tip, which has not been included in this study yet, will further complement the current soft manipulator prototype. Furthermore, pneumatic actuation is applied during current field tests, which results in a slow response time (based on the fact that we used a bunch of long pneumatic tubes) that constrains the manipulator's speed. In future studies, we will employ multi-channel hydraulic actuators with a system that can be mounted on the robot to enhance the grasping efficiency, as well as exploit a fully untethered underwater robot. In order to extend the application of this soft manipulator into the deep sea collection, we will explore the impact of water depth, oceanic current to the locomotion precision and stability in the future study. We will also apply more advanced modeling and control methods (such as the machine learning) to compensate for the system errors, and increase the grasping accuracy and dynamic response under the unstructured environment.

## Author Contributions

LW conceived the project. ZG accomplished the modeling, actuation, control, and kinematics experiments. ZG, BC, JL, XF, and ZL conducted the underwater robot system and demonstrated the underwater grasping experiments. LW and ZG prepared the manuscript, and all authors provided feedback during subsequent revisions.

### Conflict of Interest Statement

The authors declare that the research was conducted in the absence of any commercial or financial relationships that could be construed as a potential conflict of interest.
